# Supplementary material for: The potential health impact of restricting less-healthy food and beverage advertising on UK television between 05.30 and 21.00 hours: A modelling study
Source: PLoS Med. 2020 Oct 13;17(10):e1003212. doi: 10.1371/journal.pmed.1003212 (PMC7553286; doi:10.1371/journal.pmed.1003212)
Supplement: S1 Data — (DOCX) [file pmed.1003212.s001.docx]

**SUPPLEMENTARY DATA**

**Table A: Differences in BMI by social grade and age reported in the Millennium Cohort Study**

| **Age (years)** | **Difference in BMI relative to social grade AB (kg/m^2^)** | |
| --- | --- | --- |
|  | **Social grade C** | **Social grade DE** |
| 7 | 0.15 | 0.45 |
| 11 | 0.55 | 1.05 |
| 14 | 0.45 | 1.4 |

Source: Supplementary Table 1 in Bann D, Johnson W, Li L, Kuh D, Hardy R. Socioeconomic inequalities in childhood and adolescent body-mass index, weight, and height from 1953 to 2015: an analysis of four longitudinal, observational, British birth cohort studies. Lancet Public Health 2018; published online March 20. http://dx.doi.org/10.1016/ S2468-2667(18)30045-8.

**Table B: Estimates for changes in number of incident cases across the life-course for today’s children (n=13,729,021) attributable to changes in weight status arising from a 9pm watershed**

| Scenario | Ischaemic Heart Disease | Diabetes | Stroke | Cancer* | Cirrhosis |
| --- | --- | --- | --- | --- | --- |
| A | -8,800 | -54,000 | -12,000 | -4,600 | -2,000 |
| B | -2,700 | -16,000 | -3,700 | -1,400 | -630 |

*Cancer includes breast cancer, colorectal cancer, liver cancer, kidney cancer and pancreatic cancer

**Table C: Sensitivity analysis for scenario A (all HFSS advertising between 0530 and 2100 is withdrawn)**

| **Sensitivity Analysis** | **Reduction in adverts seen per day** | **Reduction in obesity** | **Reduction in overweight** | **QALY gain**  **(discounted)** | **Net monetary benefit (£million)** |
| --- | --- | --- | --- | --- | --- |
| Base scenario | 1.5 | 40,000 (12,000-81,000)  4.6% (1.4%-9.5%) | 120,000 (34,000-240,000)  3.6% (1.1%-7.4%) | 120,000 (32,000-250,000) | £7,400 (£2,000-£16,000) |
| 2018 Nutrient Profile Model | 2.0 | 52,000 (14,000-110,000)  6.1% (1.6%-12.6%) | 150,000 (40,000-320,000)  5.0 % (1.4%-11.5%) | 150,000 (43,000-340,000) | £9,700 (£2,700-£21,000) |
| Brand adverts all HFSS | 1.7 | 45,000 (13,000-99,0000)  4.1% (1.2%-9.3%) | 130,000 (37,000-300,000)  4.1% (1.1%-9.2%) | 140,000 (40,000-300,000) | £8,400 (£2,400-£18,000) |
| Brand adverts all non-HFSS | 1.3 | 35,000 (10,000-79,000)  4.1% (1.2% -9.3%) | 100,000 (29,000-230,000)  3.2% (0.9%-7.3%) | 110,000 (30,000-250,000) | £6,700 (£1,900-£16,000) |
| NICE discount rate assumed | 1.5 | 40,000 (12,000-81,000)  4.6% (1.4%-9.5%) | 120,000 (34,000-240,000)  3.6% (1.1%-7.4%) | 46,000 (13,000-110,000) | £3,100 (£980-£7,200) |
| *Differential impact by BMI status | 1.5 | 57,000 (16,000-120,000)  6.7% (1.8%-14.3%) | 170,000 (45,000-360,000)  5.2% (1.4%-11.3%) | 130,000 (38,000-290,000) | £8,100 (£2,400-£18,000) |

*To model the differential impact by BMI (i.e. for healthy and overweight children), we assumed 13.2 (0.7-25.7) kcal reduction in children with overweight and 7.3 (0.4-14.2) in children with a normal BMI.

**Table D: The effect on obesity and overweight of different assumptions about the impact of HFSS TV advertising on older children (aged 15-17 years)**

| **Scenario** | **Assumptions and parameters (for children aged 15-17 years)** | | | **Reduction in overweight and obesity for all children (5-17 years)** | |
| --- | --- | --- | --- | --- | --- |
|  | **Assumption** | **Change in adverts seen** | **Change in energy intake (kcal)** | **Obesity** | **Overweight** |
| A | Older children not affected by adverts; relative shift in BMI distribution at age 14 years continues to age 17 years | n/a | 0 | 40,000 (12,000-81,000)  4.6% (1.4%-9.5%) | 120,000 (34,000-240,000)  3.6% (1.1%-7.4%) |
| A | Older children affected by adverts child viewing pattern | - 1.5 | -9.1 (-0.5 to -17.7) | 40,000 (12,000-82,000)  4.7% (1.4%-9.7%) | 120,000 (34,000-240,000)  3.6% (1.1%-7.6%) |
| A | Older children affected – adult viewing pattern | - 4.4 | -26.7 (-1.3 to -52.0) | 59,000 (16,000-120,000)  6.9% (1.9%-14.4%) | 160,000 (43,000-340,000)  5.0% (1.3%-10.7%) |
| B | Older children not affected by adverts; relative shift in BMI distribution at age 14 years continues to age 17 years | n/a | 0 | 12,000 (3,100-28,000)  1.4% (0.4%-3.3%) | 35,000 (9,000-81,000)  1.1% (0.3%-2.5%) |
| B | Older children affected – child viewing pattern | -0.5 | -2.8 (-0.1 to -5.3) | 12,000 (3,700-28,000)  1.5% (0.4%-3.2%) | 36,000 (11,000-80,000)  1.1% (0.3%-2.5%) |
| B | Older children affected – adult viewing pattern | +0.3 | +1.5 (+0.1 to +3.0) | 7,600 (2,100-17,000)  0.9% (0.2%-2.0%) | 25,000 (6,700-55,000)  0.8% (0.2%-1.7%) |

Estimates of number of children are based on 2015 population; Scenario A: all advertising between 0530 and 2100 is withdrawn; Scenario B: all advertising between 0530 and 2100 is displaced to 2100 to 0530; estimates based IOTF = International Obesity Task Force cut-points

**Table E: Comparison with Brown et al paper from Australia**

|  | **Brown et al:**  **9.30pm watershed in Australia (Brown et al)** | **Our model:**  **9pm watershed in the UK (Scenario A)** |
| --- | --- | --- |
| **Total population** | 24 million | 65 million |
| **Age group affected** | 5-15 years | 5-14 years |
| **Food adverts viewed per child** | Not specified | 4.7 per day |
| **Advertising impact on calorie consumption** | 12.3 kcal/minute | 14.2 kcal/minute |
| **Adverts ‘removed’** | 3.6 per day (approximately) | 1.5 per day |
| **Reduction in energy intake** | 27.3 kcal/day | 9.1 kcal/day |
| **Change in mean body mass index** | -0.35 kg/m^2^ | -0.12 kg/m^2^ |
| **Discounting assumptions** | 3% for health benefits and costs | 1.5% for health benefits, 3.5% for costs; tapered discounting rates |
| **QALY/HALY (lifetime)** | 88,000 HALY (discounted at 3% p.a.) | 120,000 QALYs (discounted) |
| **Cost Savings (lifetime)** | 770 million AUD | £490 million (healthcare, formal care, productivity) |
