# Supplementary material for: The potential health impact of restricting less-healthy food and beverage advertising on UK television between 05.30 and 21.00 hours: A modelling study
Source: PLoS Med. 2020 Oct 13;17(10):e1003212. doi: 10.1371/journal.pmed.1003212 (PMC7553286; doi:10.1371/journal.pmed.1003212)
Supplement: S1 Text — (DOCX) [file pmed.1003212.s002.docx]

**Health Impact Assessment: 9pm Watershed**

**Aims:**

Primary aim: To quantify the health impact and wider benefits (social and employment that accrue) of the proposed 9pm watershed (and variants of) for unhealthy food advertising

Secondary aim: To undertake a cost-effectiveness analysis from the perspective of the state

**Methods**

General Approach

We will follow the broad approach used by Brown et al to model the health impact of a proposed 9.30pm watershed in Australia.[1]

We will assume that exposure to unhealthy food advertising on TV has a short-term (uncompensated) effect on calorie consumption, which cumulatively leads to excess calorie consumption that results in weight gain and an increase in BMI. This estimate of this effect will be based on the Russell et al systematic review.

Whilst we acknowledge there are two ‘pathways’ through which advertising affects diet, in practice the data currently only permits us to model one of these two pathways (that depicted at the top of Figure 1).

We will use the use the validated Hall equations,[2] to estimate the effect of increased calorie intake on children’s BMI.

We assume that the additional calorie intake during childhood shifts the BMI distribution curve and this relative shift is then maintained throughout life.

We will use the PRIMEtime model (Cobiac et al, University of Oxford) to estimate the health impacts (as well as health and social care costs) in adult life.

Figure 1: Pathways through which food advertising affects health


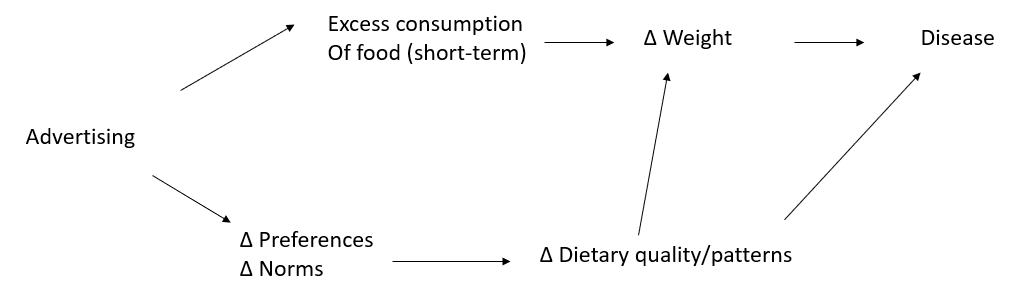


Scope

Only consider effects on children (aged 5-14 years), based on the age range included in the Russell et al meta-analysis (2-14 years); and Hall equation validity for 5 years and over.

UK wide

Benefits: health, healthcare costs, social care costs, productivity costs

Costs: costs of implementation borne by FSA-Ofcom

Scenarios modelled

[See detailed notes from IFS on the how of modelling these scenarios]

The two broad scenarios we will explore are:

1. ‘Unhealthy food advertising’ is completely removed
2. ‘Unhealthy food advertising’ shifts from pre-watershed to the immediate postwatershed slot after the watershed (9-11pm), i.e. we model a plausible industry response based on past example from 2007 regulations

In addition there are three options under consideration around the use of the nutrient profile: use the old model, use the new model; use the old model for the 9pm watershed and the new model for children’s TV programmes.

This yields six scenarios:

| A1 | Everything that is above threshold between 0530 and 2059 ‘disappears’. Based on current/old NPM |
| --- | --- |
| A2 | Everything that is above threshold between 0530 and 2059 ‘disappears’. Based on new NPM |
| A3 | Everything that is above threshold between 0530 and 2059 ‘disappears’. Based on current/old NPM. Additionally, the new NPM is used for children’s TV programmes |
| B1 | Everything that is above threshold between 0530 and 2059 moves to the immediate post-watershed slot (9pm -11pm). Based on current/old NPM |
| B2 | Everything that is above threshold between 0530 and 2059 moves to the immediate post-watershed slot (9pm -11pm). Based on new NPM. |
| B3 | Everything that is above threshold between 0530 and 2059 ‘disappears’ moves to the immediate post-watershed slot |

Our primary scenario (for consideration with respect to sensitivity analyses is A2 as this is the primary ‘ask’ from the public health community)

Health Outcomes

- Childhood obesity & overweight (prevalence, numbers)
- Disease (T2DM, IHD, stroke breast cancer, colon cancer, liver cancer, kidney cancer and cirrhosis)
- Premature mortality prevented
- QALY gain/loss (in adult life, tbc child life)

Economic Analyses

Economic costs/revenue

- Health care costs
- Social care costs (‘formal care costs’)
- ‘production costs’ (?employment/productivity)
- ‘all other societal costs’
- Costs of monitoring/enforcement

As is custom in the UK we will not include the costs of passing the legislation.

This will enable the following economic analyses (? Done on scenarios A1-3 and B1-3; or just A or just A2):

- Cost effectiveness analysis (Healthcare costs vs intervention costs)… so that this is comparable with other PH/healthcare analyses this should be done using NICE rules
- Net monetary benefit (health care + social care + productivity vs known intervention costs) + as part of this undertake a threshold analysis; using Treasury rules; should we display the costs/benefit both discounted and undiscounted?

|  | Treasury | NICE |
| --- | --- | --- |
| QALY | 60k | 20k |
| Discount rate QALY | 1.5% | 3.5% |
| Discount rate costs | 3.5% | 3.5% |

From the Treasury Green Book, we will assume a 3.5% discount for costs (including social care costs), but benefits (QALY) discounted at 1.5% for the first 30 years then the discount rates declines, as set out on p.104.

Modelling the (acute) impact of TV food advertising on calorie consumption

Based on the Russell et al meta-analysis: exposure to TV advertising (59.4 kcals for 4.23 minutes 🡪 14.0 kcal/minute, CI lower 0.7 and CI upper 27.) [3]

Russel et al meta-analysis has been chosen as the most recent and most comprehensive (see Table 1); we will only include studies of TV advertising and those that report the duration of advertising exposure (n=8).

Other studies considered are shown in Table 1; we will formally tabulate these estimates and if there is marked heterogeneity consider a sensitivity analysis where we use a different value.

Children are watching (depending on which are considered the unhealthy adverts) 3-6 adverts per day, on the basis of 25.9s per advert (Weighted by child impacts: a median of 30  secs; mean 25.9, standard deviation 11.9) this equates to 1.5 to 3 minutes per day; thus in the range we are looking at which falls within the 4.23 minutes of the meta-analytic studies we will assume a linear relationship

We will model an average effect, and assume the same effects for children of all ages (5-14 years)

Modelling change in calories on weight status

- Assume a mean calorie change will affect BMI using the Hall equations
- The BMI curve is plotted as a log normal curve (reflecting a right skew)
- We assume the position of the curve shifts, i.e. the median BMI shifts based on the mean calorie change; the variance of the curve is unaltered

Exposure to TV advertising amongst children

- Based on BARB dataset (2015, children aged 4-15 years; UK)
- Reports total impacts by time of day (one impact = one child watching an advert; 10 impacts = 10 children watching one advert, or 1 child watching 10 adverts, etc)
- Adverts are classified into above NPM threshold, below NPM threshold and uncertain (restaurants & bars, supermarkets, brand adverts)
- We will use the current NPM model; and the revised model (the one consulted in 2018)
- For base scenario we will assume that restaurants & bars (mostly fast-food) is above threshold, a proportion of brand adverts (based on knowledge of the brand is above threshold), and supermarkets are below threshold)
- Assume advert duration = 22.7 s (mean from Nielsen dataset, 2015)

Sensitivity analyses (which we run just on scenario A2)

1. **Classification of brand adverts** (our primary assumption is that some are above and some below; and we run a sensitivity analysis with i) all adverts being treated above, and ii) all adverts being treated below)
2. **Older children:** assume advertising has the same effect on children who are aged 15-18 years; and assume their advertising exposure is like children or like adults [we need to run this on scenario A and B]
3. **Tracking of obesity into adult life:** No tracking of benefits into adult life
4. **Differential impact of advertising by weight status:** assume the variance of the curve (i.e. its shape changes) as well as its position, this will have the effect of assuming that children who are overweight and obese are more affected by the policy as shown in the Russell et al meta-analysis.[3]
5. **Impact of obesity on health: no benefit assumed age 15 years and above;** – i.e. this means that no disease benefits accrue in later life; basically all the measured health benefit is coming from the effects on weight-related impacts on quality of life in childhood
6. **More conservative assumption around impact of advertising on calorie consumption:**

Reduction factor for application of experimental effect of advertising on calorie consumption to real-world setting; 0.50 (95% UI 0.16–0.85); Sampled from a pert distribution, minimum 0.00, most likely 0.50, maximum 1.00. As used by Brown. [Brown et al also used an adjustment factor for compensation at mealtimes based on Cecil et al 2005,[4] but given this research has now been superseded Norman et al,[5] and no compensation effect found we will not model this effect]

1. **No lags in population impact fraction** (at the moment we assume a 5 year lag in effect on cardiovascular disease and 20 year lag on cancers)

**References**

1. Brown V, Ananthapavan J, Veerman L, Sacks G, Lal A, Peeters A, et al. The Potential Cost-Effectiveness and Equity Impacts of Restricting Television Advertising of Unhealthy Food and Beverages to Australian Children. Nutrients. Multidisciplinary Digital Publishing Institute (MDPI); 2018;10. doi:10.3390/nu10050622

2. Hall KD, Butte NF, Swinburn BA, Chow CC. Dynamics of childhood growth and obesity: development and validation of a quantitative mathematical model. Lancet Diabetes Endocrinol. Elsevier; 2013;1: 97–105. doi:10.1016/S2213-8587(13)70051-2

3. Russell SJ, Croker H, Viner RM. The effect of screen advertising on children’s dietary intake: A systematic review and meta-analysis. Obes Rev. 2018; doi:10.1111/obr.12812

4. Cecil JE, Palmer CN, Wrieden W, Murrie I, Bolton-Smith C, Watt P, et al. Energy intakes of children after preloads: adjustment, not compensation. Am J Clin Nutr. 2005;82: 302–308. doi:10.1093/ajcn.82.2.302

5. Norman J, Kelly B, McMahon A-T, Boyland E, Baur LA, Chapman K, et al. Sustained impact of energy-dense TV and online food advertising on children’s dietary intake: a within-subject, randomised, crossover, counter-balanced trial. Int J Behav Nutr Phys Act. BioMed Central; 2018;15: 37. doi:10.1186/s12966-018-0672-6

6. Veerman JL, Van Beeck EF, Barendregt JJ, Mackenbach JP. By how much would limiting TV food advertising reduce childhood obesity? Eur J Public Health. Oxford University Press; 2009;19: 365–369. doi:10.1093/eurpub/ckp039
