# Supplementary material for: The potential health impact of restricting less-healthy food and beverage advertising on UK television between 05.30 and 21.00 hours: A modelling study
Source: PLoS Med. 2020 Oct 13;17(10):e1003212. doi: 10.1371/journal.pmed.1003212 (PMC7553286; doi:10.1371/journal.pmed.1003212)
Supplement: S2 Text — (DOCX) [file pmed.1003212.s003.docx]

**Changes to analytic plan**

**Aims:**

The primary aim was restricted to focus on health impact as this was what the model was best able to address.

We could not identify any costs from the perspective of the state, instead we have just given monetised estimate of the health benefits.

Scenarios modelled

We chose to reduce the number of scenarios from six to two for simplicity, given the revised nutrient profile model was still awaiting approval for use. We did, however, undertake a sensitivity analysis around the use of this new model.

Health Outcomes

To simplify and rationalise the presentation of results we chose to omit premature mortality as an outcome.

Sensitivity analyses

**The sensitivity analysis for “tracking of obesity into adult life:** No tracking of benefits into adult life,” was not explicity run as it is effectively the same as assuming no impact on DALYs in adult life. This information is effectively captured in the output that estimates the DALYs in childhood.

**More conservative assumption around impact of advertising on calorie consumption:** we did not undertake this analysis as there is no strong empirical basis to guide how this should be done.

**No lags in population impact fraction:** to rationalise the number of results we dropped this analysis, in part, because it was considered uninformative given that all the changes in BMI occur during childhood.

Estimates of by SES

In response to peer review comments we additionally looked at the impacts (on obesity) by SES. This supported a shift in focus to a health impact study – rather than a health impact and cost-effectiveness study.
