# Supplementary material for: The potential health impact of restricting less-healthy food and beverage advertising on UK television between 05.30 and 21.00 hours: A modelling study
Source: PLoS Med. 2020 Oct 13;17(10):e1003212. doi: 10.1371/journal.pmed.1003212 (PMC7553286; doi:10.1371/journal.pmed.1003212)
Supplement: Attachment — Submitted filename: Response to reviewers.docx [file pmed.1003212.s004.docx]

Title- please ensure the title entered into EM matches the title of your study. I believe the 2 after “beverage” is a typo

R: Thank you – we have corrected.

Abstract

Please structure your abstract using the PLOS Medicine headings (Background, Methods and Findings, Conclusions). * Please combine the Methods and Findings sections into one section, “Methods and findings”.

R: Thank you. We have modified this as you suggest.

Abstract Methods and Findings: * Please ensure that all numbers presented in the abstract are present and identical to numbers presented in the main manuscript text. *

Please include the study design, population and setting, number of participants, years during which the study took place, length of follow up, and main outcome measures. * Please quantify the main results (with 95% CIs and p values). * Please include the important dependent variables that are adjusted for in the analyses. * * In the last sentence of the Abstract Methods and Findings section, please describe the main limitation(s) of the study's methodology.

Conclusions- please begin with “our results show” or similar

* Please address the study implications without overreaching what can be concluded from the data; the phrase "In this study, we observed ..." may be useful. * Please interpret the study based on the results presented in the abstract, emphasizing what is new without overstating your conclusions. * Please avoid vague statements such as "these results have major implications for policy/clinical care". Mention only specific implications substantiated by the results.

R: The abstract has been modified to reflect these requests.

Data Availability Statement

The Data Availability Statement (DAS) requires revision. For each data source used in your study: a) If the data are freely or publicly available, note this and state the location of the data: within the paper, in Supporting Information files, or in a public repository (include the DOI or accession number). b) If the data are owned by a third party but freely available upon request, please note this and state the owner of the data set and contact information for data requests (web or email address). Note that a study author cannot be the contact person for the data. c) If the data are not freely available, please describe briefly the ethical, legal, or contractual restriction that prevents you from sharing it. Please also include an appropriate contact (web or email address) for inquiries (again, this cannot be a study author).

R: We have inserted this at the end of the manuscript. We were unclear whether this should be sited there or in the methods, either at the end of the methods or integrated into the methods. Please advise.

Author Summary

At this stage, we ask that you include a short, non-technical Author Summary of your research to make findings accessible to a wide audience that includes both scientists and non-scientists. The Author Summary should immediately follow the Abstract in your revised manuscript. This text is subject to editorial change and should be distinct from the scientific abstract. Please see our author guidelines for more information: <https://journals.plos.org/plosmedicine/s/revising-your-manuscript#loc-author-summary>

R: We have inserted an author summary after the abstract.

References

Please update the bibliography to Vancouver style and place all full stops after square brackets within text. For example xxx [1].

R: References are Vancouver style and stops are now after the bracket.

Methods

Please remove the section entitled “ethics and public involvement”

R: This has been removed.

Please briefly provide demographic information for the 5100 homes used in this study

R: Unfortunately, BARB do not provide a specific breakdown, but they do state “the BARB panel is representative of television viewing across the country in terms of demography, geography, ethnicity and TV platform. It is not possible to volunteer to join the panel; households are selected randomly. The design of our panel includes targets for the number of homes of different types. Of course, no panel can be perfectly representative, so we also weight our data. Our panel targets and weighting are derived from UK Government census categories and population figures, to ensure that the panel is representative of all sectors of the population.”

We have amended the text to: “BARB calculated the number of child impacts by using a panel of 5,100 homes. The panel is reported to be representative of the UK in terms of demography, geography, ethnicity and TV platform, but BARB do not provide a specific breakdown”

Line 257-258-please report these data or remove this reference

R: We think there may be a misunderstanding, the data that is not reported is data that is not reported by the Millennium Cohort study, not data that we did not report (we do report the data in the supplementary information). We have changed this sentence to read: “We used linear interpolation and extrapolation to estimate differences in BMI for age groups for which data was not directly reported from this [i.e. Millennium Cohort] study. Our estimates are shown in the supplementary material.”

Please ensure that the study is reported according to the STROBE guideline, and include the completed STROBE checklist as Supporting Information. When completing the checklist, please use section and paragraph numbers, rather than page numbers. Please add the following statement, or similar, to the Methods: "This study is reported as per the Strengthening the Reporting of Observational Studies in Epidemiology STROBE guideline (S1 Checklist)." Please report your study according to the relevant guideline, which can be found here: <http://www.equator-network.org/>

R: There are no reporting guidelines for public health modelling studies, and previously when publishing in PLOS Medicine we have not used STROBE (or other) reporting guidelines. The generic items (title, abstract, introduction, discussion and funding) work fine, but many of the specific items – particularly around results are not applicable.

Did your study have a prospective protocol or analysis plan? Please state this (either way) early in the Methods section. a) If a prospective analysis plan (from your funding proposal, IRB or other ethics committee submission, study protocol, or other planning document written before analyzing the data) was used in designing the study, please include the relevant prospectively written document with your revised manuscript as a Supporting Information file to be published alongside your study, and cite it in the Methods section. A legend for this file should be included at the end of your manuscript. b) If no such document exists, please make sure that the Methods section transparently describes when analyses were planned, and when/why any data-driven changes to analyses took place. c) In either case, changes in the analysis-- including those made in response to peer review comments-- should be identified as such in the Methods section of the paper, with rationale

R: We have added two supplementary files: analytic plan; changes to analytic plan. We have referenced these at the end of the first paragraph of the methods.

Comments from the reviewers:

Reviewer #1: This paper aims to estimate the health impact of prohibiting the advertising of food and beverages high fat sugar and salt (HFSS) from 0530 to 2100 on television in the UK. This is valuable research in light to reducing childhood obesity and overweight by 2030. Although methodology is complex it is well explained and accurately described. This paper will be of particular interest to those that participate in general strategies for preventing obesity implemented by any government.

Minor issues

Abstract

Abstract does not follow the author's guideline: Background + Methods and Findings + Conclusions

R: Thank you – now amended to reflect this structure.

Major issues

Methodology

Methodology is complex but it is well explained and accurately described. Figure 1 (line 107) helps to understand the whole process.

Television audience data is from 2015 (line 138). We understand the difficulty in finding current open data, but this fact damages the quality of the research and the final conclusions. Companies like Nielsen are used to transfer their data for free for university research, authors should ask for it.

R: Whilst we agree more recent data would aid this study (albeit the context is likely to have changed since the start of the pandemic), it is not our experience that the data is freely and readily available. We had to pay for this dataset – and despite repeated attempts by different members of the team have never been able to access a granular dataset (that would be necessary for this type of analysis) from Nielsen.

It should be noted that television consumption by children in the UK was 97% in 2015 but fell to 72% in 2018, according to Ofcom (Children and Parents: Media Use and Attitudes Report 2018, p. 28). Multi-screen consumption has not been taken into account, it occurs when watching television using the smartphone or tablet at the same time. The impacts that come from digital media are impossible to control and it would be an important limitation. Ofcom's study states that 37% of children aged 5-15 use smartphones.

R: We agree and state the following in the limitations: “We have only considered one form of advertising media (television), in part reflecting the absence of good data on exposure to advertising on other media or its effects on consumption. Children’s television viewing has declined in recent years, and shifted towards digital viewing[56]. Nonetheless currently television viewing is likely to be an important source of exposure to video-based advertisements[57]. If the downward trend in television exposure is associated with a downward trend in HFSS advertising exposure (i.e. assuming the pattern, frequency and duration of advertising has not changed) and it were to continue, then our results are likely to have overestimated the impact of television advertising restrictions on children’s weight status.”

Whilst we have not explicitly explored on-line video advertising (which we cannot quantify), the exposure may well be similar, and thus our study could be seen as adding further weight to the need to regulate this space. Under policy implications, we do state the following: “Our findings suggest that television advertising of less-healthy food, and likely other similar advertising (i.e. video advertising online), makes a meaningful contribution to increasing levels of childhood obesity. Thus, measures that successfully reduce children’s exposure to less-healthy food advertising are likely to make a valuable contribution to the government’s goal of halving childhood obesity by 2030.”

Advertising exposure has been measured by impacts (line 140). It would be advisable to have information about the audience in thousands of individuals who have been impacted. It is important to distinguish between impacting a large percentage of these children a few times or impacting the same group many times. It is an important fact to calculate the decrease in childhood obesity.

R: Whilst there will be a distribution, we know that most children watch some television (96% in 2015). We are much closer to the scenario of impact a large percentage of children a few times than a few children many times.

We have modified the methods to read as follows: “We assumed the average impact seen by a child in this dataset (defined as aged 4-15 years) was the average impacts seen by a child aged 5-14 years, i.e. that impacts were spread widely across the population, given that 96% of children aged 5-15 years are reported to watch television in the UK [35].”

However, we are undertaking population-level modelling, and the overall impact for population health is generally no different. For population health changes in the overall numbers of people with obesity or overweight can be the same whether it is a few people who experience a large change in BMI or a small change for everybody. There are some circumstances when distribution does matter for overall estimates, principally when different distributions interact, e.g. if children who have excess weight watch more (or less television). We have explicitly explored this scenario in one of the sensitivity analyses.

We have also inserted new text to the limitations (under advertising data): “Impacts as a measure does not contain information on the distribution of television viewing within a population, and we assumed a uniform distribution (i.e. all children watch the same amount of television). Given that most children (96%) watch television,[35] it seems likely that many children will be affected by these measures by a small amount rather than a few children being affected by a very large amount. We have also explored scenarios under which TV viewing is not uniform, but varies by social grade and weight status.”

On line 212 it is said that if television minutes are reduced, the calories consumed will be reduced. Do you have any data that shows that a 20 second ad is less effective than a 30? Sometimes even 10 second ads are more effective because they are based on specific promotions that are very successful among children. Possibly the number of ads is more important than the exposure time.

R: The Russell et al meta-analysis (that we use) found a non-significant trend between advertising duration and calorie intake. We used a linear approach as it has been used by others, it is the simplest relationship to model (and one that is commonly observed), and has some empirical support (albeit weak). Nonetheless, we agree our approach to modelling the relationship between advertising and calorie intake is a limitation and assuming different relationships could yield different results. We state: “We have assumed a linear relationship between cumulative advertising exposure (measured in time) and calorie consumption. We have not explicitly explored other relationships (e.g. assuming non-linear relationships or accounting for frequency of exposure), which may affect the outcomes”

It is assumed that television consumption by social class follows the same guidelines as in 2009 (line 242). Some new research associate lowest social class with a higher consumption of internet and digital devices by children. This information should be updated.

R: Our focus is on television, not digital devices. Internet and digital devices can be used in different ways, e.g. they can be used as educational tools or communication devices – with some parents using ad blocking software (the patterning of which is unknown, but might conceivably be used more extensively by parents in higher social grade).

Reviewer #2: Referee report on "Quantifying the potential health impact of restricting less-healthy food and beverage advertising on UK television between between 0530 and 2100: a modelling study (PMEDICINE-D-19-04447)

The paper provides an assessment on the potential impact of restricting advertising of less healthy food on UK television. A key novelty is the use of advertising data with information on timing and contents of adverts. The paper does not provide new empirical estimates of the effects of the policy on behaviour (and other subsequent outcomes), but combines the above information on advertising with estimates from previous literature on (i) the effect of advertising exposure on calorie intake by children; (ii) the associated weight gain; as well as related (iii) health effects and (iv) effects on healthcare costs. Due to uncertainty regarding producer and advertiser reactions to stricter regulation, the paper compares the effects of two different scenarios, one where all adverts within the specified time frame are withdrawn, and one where all adverts are displaced to slots outside the specified time frame.

I am sympathetic to the topic of the paper, and view efforts of reducing childhood obesity to be of major societal importance. The paper is clearly written and well-organized. My main questions relate to whether the value added vis-à-vis earlier literature, and the new information generated by the paper are sufficient to warrant publication in PLOS Medicine. Below, I provide some comments and questions:

Main comments:

1)Contribution of the paper. Is the value added / contribution of the paper sufficient for this journal, compared e.g. to the previous Australian study on the same topic? a key argument made by the authors is that advertising and dietary habits of UK children may differ from those found in Australia; but, given this argument, is this just another country specific study, and as such is the contribution sufficient for this journal? Coming from a different discipline, I am ill-equipped to make this judgement, but nevertheless think that this question should be raised.

The use of genuine and detailed data on advertising is a definite strong point, but is using such data a sufficient novelty. The paper argues that exploring the potential impact of industry's response to the policy, and analyzing effects by SES, are additional contributions. These do not appear to be a key focus however. The potential industry response is only taken into account through comparing the two hypothetical scenarios; the result of the comparison is quite obvious (displacement leading to smaller effects than complete withdrawal). Examining effects by SES is certainly of interest, but necessarily subject to a lot of uncertainty and simplifying assumptions. The point estimates for the different outcomes (table 4) differ slightly (in an intuitive way), but the confidence intervals are very wide and largely overlapping, so not much can be said / learnt in the end about differences across SES.

To put it bluntly, we don't really learn much regarding differences between

SES (we cannot say with any confidence whether there are any differences, and how large they might be).

R: We do think our work makes an important and unique contribution. Whilst the context is the UK, some of the messages and findings have broader relevance, given the widespread interest in the topic. Nearly all public health policies will be (or at least appear) context specific. Scientific advancement depends on comparing, contrasting and aggregating data over a set of contexts (hence the value of comparison with Australia) from which more generalisable inference may be drawn. Besides its policy value there are also methodological differences and extensions compared to Australia.

We suggest this reviewer’s interpretation of the differences by SES is too conservative. The sources of error are likely to largely be similar across SES groups, so if the true estimate for group AB is towards the higher end of the range, then the same pattern would be expected for the other two social grade groups. So we suggest we can be relatively confident of the differences and have an indicative estimate of the relative differences.

2) Taking into account uncertainty. I would like to see more of a discussion on how uncertainty inherent in combining estimates (that are themselves subject to large uncertainty) from various different sources is taken into account. Do you account for uncertainty inherent in all the steps (i) - (iv) above, each involving externally estimated parameters, and how? Already the CI in the first step - effect of an extra minute of advertising exposure on calorie intake - is very large (0.7 to 27.7 kcal per day), and similar uncertainty is likely in the other steps. More details should be given here, to understand whether the CI reported are correct / what assumptions are made, and how they should be interpreted.

R: We have extended this description (see methods – uncertainty intervals), and it now reads: “We determined uncertainty (95% uncertainty intervals) for our main outcome measures using Monte Carlo analysis. To do this we run the model 1000 times, for each run we use a different estimate for each parameter, drawn from the distribution described by the parameter’s mean and standard deviation.”

Other comments:

3)	Related to point #1, the paper refers to differences between tv viewing patterns and advertising practices between UK and Australia. Is it possible to briefly summarize what is known about these differences.

R: This is summarised in the supplementary material (Table 5) – and signposted in the discussion.

4)	There is a slight contradiction in the text on lines 143-144 "two impacts can represent two different people viewing the same advert once, or one person viewing two different adverts" vs. lines 177-178 "two impacts could refer to two different people viewing an advert once, or one person viewing an advert twice". This is probably just a typo, but should be clarified; viewing the same advert twice probably has a different impact than viewing two different adverts.

R: Thank you. This has been amended to say “one person viewing the same advert twice”

5)	Can we think of scenario A as providing an upper bound for the effects of the policy, while scenario B provides a lower bound? if so, it would be helpful to mention this.

R: Thank you for this suggestion. We have amended the policy implications section of the discussion to read. “For this reason, we suggest the modelled scenarios are best understood as explorations of what the policy could achieve rather than predictions of what will happen; the two scenarios capturing the range of responses that might be expected, at one extreme no industry adaptation and at the other maximum shifting of advertising.”

6)	Persistence of weight effects. The paper writes that "we assumed the shift in the BMI curve that we observed at age 15 years persisted into later life". This is explained only on p. 16, where you say that you assume that exactly the same absolute change in BMI persists for the entire life of the person. This should be clarified earlier. Also, is it reasonable (there's certainly a correlation in BMI over lifetime, but is this particular assumption justified, and how important is it for the results)?

R: We agree this is an important part of the methods. It is mentioned on page 8 (in the methods), it should also be remembered that the persistence in to adult life is only relevant for estimating DALY and not for childhood BMI, overweight and obesity.

We agree – and discuss this issue thoroughly in the discussion, the (slightly extended) text reads: “We have assumed that the relative change in mean body mass index associated with change in exposure to HFSS television advertising is maintained through life, i.e. if mean BMI is reduced by 0.1 kg/m^2^ at age 14 years, when this cohort reaches the age of, e.g. 40 years, the mean BMI is reduced by 0.1 kg/m^2^ compared to the counterfactual. It is unclear to what extent this is true, although we note weight status tends to track from childhood to adult life, and that populations with higher prevalence of obesity in childhood tend to have a higher prevalence of obesity in adult life[37,38]. Despite this we note that a significant benefit (around 20% of DALYs averted), albeit discounted benefit, occurs in childhood. This effectively provides a lower estimate of the DALYs averted, if one assumed an extreme scenario of no persistence of change in weight status from childhood to adult life.”

7)	Assumed lag of health effects. This was unclear to me: "Given uncertainty about the immediacy of the effect of change in BMI on disease outcomes, we conservatively assumed a five-year lag for cardiovascular disease and diabetes and a twenty-year lag for all cancers". Given also the assumption about persistence of weight effects, what does this mean? if a child is exposed to advertising e.g. at 10 years of age, or throughout childhood between 4 - 15 years of age, what is assumed about the occurrence of health effects?

R: Thank you. This simply means past BMI rather than present BMI is used to estimate a risk for these outcomes. We include this for completeness, but because we are not modelling changes in obesity in late life, this will not affect the comparative outcomes from our model. The sentence has been changed to read: “Given uncertainty about the immediacy of the effect of change in BMI on disease outcomes, we assumed a five-year lag for cardiovascular diseases and diabetes (i.e. mean BMI over the past five-years, rather than present BMI determined risk of cardiovascular disease) and a twenty-year lag for all cancers”

8)	"In addition to obesity-related disease, we simulated the increased burden of all other diseases in added years of life" - this is quite vague, what exactly is done here?

R: Thank you. We have tightened this up to read as follows: In addition to obesity-related disease, we simulated changes in the burden of all other diseases arising from changes in life expectancy, by making allowance for disutility associated with age that is not attributed to the diseases specifically included the model.

Typos:

-	p. 3, line 90: "health" should be "health impacts"

-	p. 4, line 108: "effects" should be "affects"

-	p. 7, line 185: "is" should be "are"

R: Thank you. All changed.

Reviewer #3: This is a superbly well written and thorough manuscript.

The study design, data sources and methods used are concisely and transparently described.

The statistical techniques and mathematical modelling applied seem to be fit for purpose.

The distribution and statistical assumptions are clearly stated and appear to be reasonably applied.

The authors have performed various sensitivity analyses for key model assumptions, and have accounted for and communicated uncertainty in their results.

The tables and figures are understandable and informative.

The results and conclusions are presented and discussed fairly, and accurately represent the findings of the model.

The limitations are discussed in detail.

All queries and comments I made during my statistical review were subsequently and adequately answered later on in the manuscript, albeit some being raised in the limitations section or covered in the supplementary document.
